# (3-Allyl­oxypicolinato-κ^2^ *N*,*O* ^2^)bis­[3,5-difluoro-2-(2-pyrid­yl)phenyl-κ^2^C^1^,*N*]iridium(III)

**DOI:** 10.1107/S1600536809046479

**Published:** 2009-11-11

**Authors:** Yu-Ling Zhao, Jing Meng

**Affiliations:** a School of Chemical and Biological Engineering, Lanzhou Jiaotong University, Lanzhou 730070, People’s Republic of China

## Abstract

The title complex, [Ir(C_11_H_6_F_2_N)_2_(C_9_H_8_NO_3_)], consists of one Ir^III^ ion, two *C*,*N*-bidentate 3,5-difluoro-2-(2-pyrid­yl)phenyl (F_2_ppy) ligands and one *N*,*O*-bidentate 3-allyl­oxypicolinate (pic-3-Oall) ligand. The Ir^III^ ion is hexa­coordinated by two C atoms and two N atoms from the F_2_ppy ligands and one N atom and one carboxyl­ate O atom from the pic-3-Oall ligand, displaying a distorted octa­hedral geometry. In the crystal structure, weak inter­molecular C—H⋯F and C—H⋯O hydrogen bonds link the complex mol­ecules into a three-dimensional supra­molecular structure.

## Related literature

For general background to phospho­rescent materials, see: Baldo *et al.* (1998 ▶, 2000 ▶); Liang *et al.* (2006 ▶); Thompson (2007 ▶); Tsuboyama *et al.* (2003 ▶). For bond lengths in organic compounds, see: Allen *et al.* (1987 ▶).
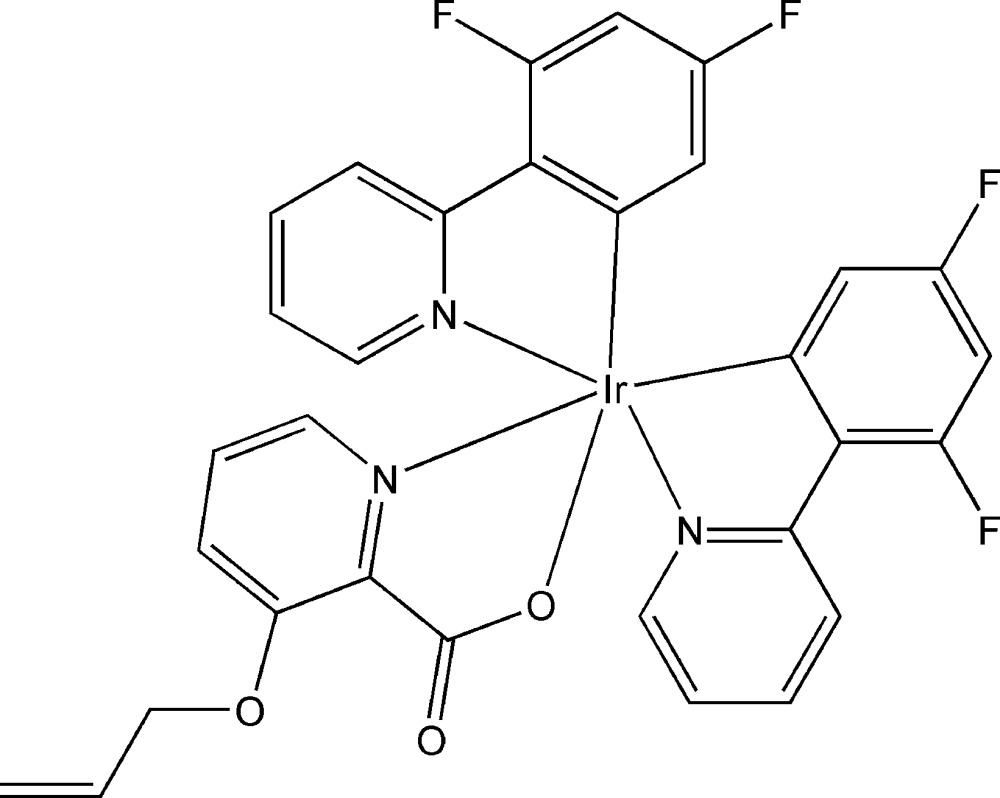


## Experimental

### 

#### Crystal data

[Ir(C_11_H_6_F_2_N)_2_(C_9_H_8_NO_3_)]
*M* *_r_* = 750.70
Monoclinic, 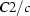

*a* = 33.429 (4) Å
*b* = 9.9117 (12) Å
*c* = 16.0265 (19) Å
β = 94.107 (2)°
*V* = 5296.5 (11) Å^3^
*Z* = 8
Mo *K*α radiation
μ = 5.11 mm^−1^
*T* = 185 K
0.31 × 0.23 × 0.03 mm

#### Data collection

Bruker SMART APEX CCD diffractometer
Absorption correction: multi-scan (*SADABS*; Sheldrick, 1996 ▶) *T*_min_ = 0.305, *T*_max_ = 0.862
14169 measured reflections
5087 independent reflections
4364 reflections with *I* > 2σ(*I*)
*R*_int_ = 0.039

#### Refinement

*R*[*F* ^2^ > 2σ(*F* ^2^)] = 0.031
*wR*(*F* ^2^) = 0.080
*S* = 1.05
5087 reflections
379 parameters
H-atom parameters constrained
Δρ_max_ = 1.72 e Å^−3^
Δρ_min_ = −0.59 e Å^−3^

### 

Data collection: *SMART* (Bruker, 2007 ▶); cell refinement: *SAINT* (Bruker, 2007 ▶); data reduction: *SAINT*; program(s) used to solve structure: *SHELXS97* (Sheldrick, 2008 ▶); program(s) used to refine structure: *SHELXL97* (Sheldrick, 2008 ▶); molecular graphics: *SHELXTL* (Sheldrick, 2008 ▶) and *Mercury* (Macrae *et al.*, 2006 ▶); software used to prepare material for publication: *SHELXTL*.

## Supplementary Material

Crystal structure: contains datablocks global, I. DOI: 10.1107/S1600536809046479/hy2251sup1.cif

Structure factors: contains datablocks I. DOI: 10.1107/S1600536809046479/hy2251Isup2.hkl

Additional supplementary materials:  crystallographic information; 3D view; checkCIF report

## Figures and Tables

**Table 1:** Selected bond lengths (Å)

Ir1—C7	1.983 (4)
Ir1—C18	2.002 (4)
Ir1—N1	2.045 (3)
Ir1—N2	2.051 (4)
Ir1—N3	2.132 (3)
Ir1—O1	2.135 (3)

**Table 2:** Hydrogen-bond geometry (Å, °)

*D*—H⋯*A*	*D*—H	H⋯*A*	*D*⋯*A*	*D*—H⋯*A*
C3—H3⋯F1^i^	0.95	2.60	2.997 (6)	106
C28—H28⋯F2^ii^	0.95	2.55	3.280 (6)	134
C10—H10⋯F3^iii^	0.95	2.50	3.299 (6)	142
C12—H12⋯F4^iv^	0.95	2.56	3.130 (6)	119
C26—H26⋯O1^v^	0.95	2.63	3.519 (5)	155
C14—H14⋯O2^vi^	0.95	2.54	3.275 (6)	134

Symmetry codes: (i) 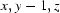; (ii) 
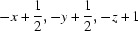
; (iii) 
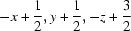
; (iv) 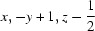; (v) 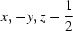; (vi) 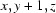.

**Fig. 1.**
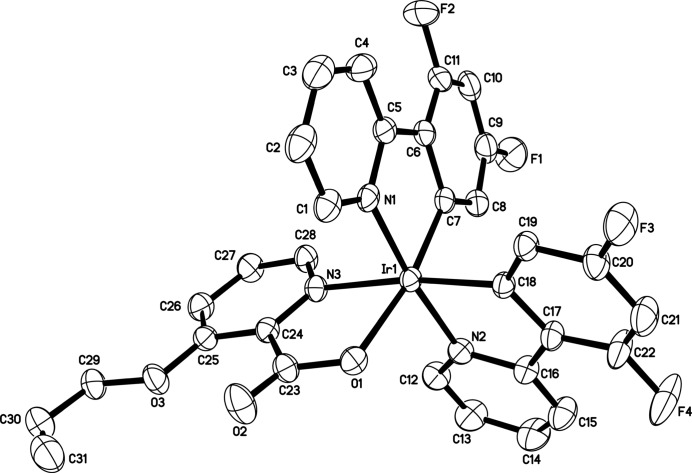
The molecular structure of the title complex. Displacement ellipsoids are drawn at the 30% probability level. H atoms have been omitted for clarity.

**Fig. 2.**
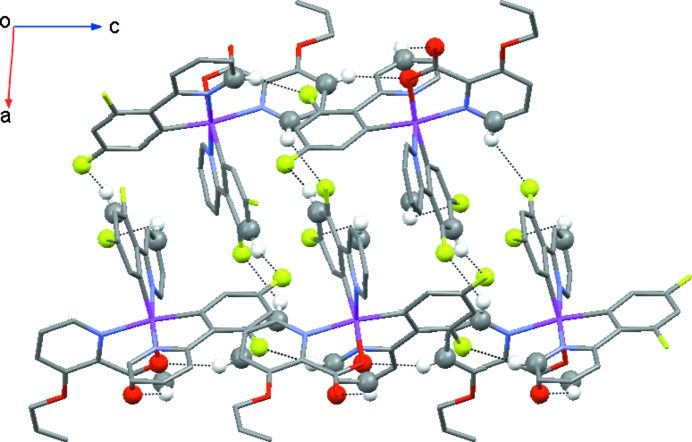
Intermolecular C—H···F and C—H···O hydrogen bonds in the title complex, viewed along the b axis. H atoms not involved in hydrogen bonds have been omitted for clarity. [Colour codes: pale-green F; grey C; red O; white H.]

## supplementary crystallographic information

### Comment

Since Forrest and co-workers (Baldo *et al.*, 1998) successfully utilized the phosphorescent material PtOEP to fabricate light-emitting devices, many heavy-metal complexes have been extensively investigated in highly efficient electrophosphorescent organic light-emitting diodes (Baldo *et al.*, 2000; Liang *et al.*, 2006; Thompson, 2007). Among the complexes, the cyclometalated iridium complexes are the most valuable emitting materials due to the results in higher efficiency and brightness (Liang *et al.*, 2006; Tsuboyama *et al.*, 2003). Recently, we synthesized a mixed-ligand iridium complex, [Ir(F_2_ppy)_2_(pic-3-Oall)] (F_2_ppy = 3,5-difluoro-2-(2-pyridyl)phenyl; pic-3-Oall = 3-allyloxypicolinate), which exhibits bright blue light.

The molecular structure of the title complex is shown in Fig. 1. The mononuclear iridium(III) complex has an approximately octahedral coordination geometry. The Ir^III^ ion is hexa-coordinated by two C atoms and two N atoms from two C,N-bidentate F_2_ppy ligands, which exhibit *cis*-C,C and *trans*-N,N chelate dispositions, and one N atom and one carboxylate O atom from one N,O-bidentate pic-3-Oall ligand. All the bond lengths and angles of the molecule are within normal ranges (Allen *et al.*, 1987). For the F_2_ppy ligands, the Ir—C bonds [1.983 (4) and 2.002 (4) Å] are shorter than the Ir—N bonds [2.045 (4) and 2.051 (4) Å] (Table 1). Due to steric interactions, the difluorophenyl groups are not coplanar with the pyridine groups; the dihedral angles between the substituted phenyl rings and pyridines are 7.60 (1) and 6.05 (3)°, respectively. For the pic-3-Oall ligand, the bond lengths of Ir—N and Ir—O are 2.132 (3) and 2.135 (3) Å, respectively. In the crystal structure, the complex molecules are connected by weak intermolecular C—H···F and C—H···O hydrogen bonds (Table 2 and Fig. 2), forming a three-dimensional supramolecular structure.

### Experimental

The title complex was prepared as following. First, a cyclometalated Ir^III^ µ-chlorobridged dimer, [(C_11_H_6_F_2_N)_2_Ir(µ-Cl)]_2_, was synthesized by reacting IrCl_3_.nH_2_O (3.0 mmol) and 2-(2,4-difluorophenyl)pyridine (7.5 mmol). Then, the dimer (0.2 mmol) was reacted with 3-hydroxypicolinic acid (0.44 mmol) in 2-methoxyethanol (25 ml), affording bis[2-(2,4-difluorophenyl)pyridine](3-hydroxypicolinate)iridium(III), which (0.2 mmol) was reacted with 3-bromopropene (0.24 mmol) in the present of anhydrous K_2_CO_3_ (1 mmol) to give the title complex. Yellow plate single crystals of the complex were obtained by slow evaporation of the chloroform solution at room temperature.

### Refinement

H atoms were positioned geometrically and refined as riding atoms, with C—H = 0.99 Å for methylene and 0.95 Å for other H atoms and with *U*_iso_(H) = 1.2*U*_eq_(C). The highest residual electron density was found 0.05 Å from Ir1 and the deepest hole 1.57 Å from C7.

### Crystal data

**Table:**

[Ir(C_11_H_6_F_2_N)_2_(C_9_H_8_NO_3_)]	*F*(000) = 2912
*M**_r_* = 750.70	*D*_x_ = 1.883 Mg m^−^^3^
Monoclinic, *C*2/*c*	Mo *K*α radiation, λ = 0.71073 Å
Hall symbol: -C 2yc	Cell parameters from 7056 reflections
*a* = 33.429 (4) Å	θ = 2.4–25.8°
*b* = 9.9117 (12) Å	µ = 5.11 mm^−^^1^
*c* = 16.0265 (19) Å	*T* = 185 K
β = 94.107 (2)°	Plate, yellow
*V* = 5296.5 (11) Å^3^	0.31 × 0.23 × 0.03 mm
*Z* = 8	

### Data collection

**Table:**

Bruker SMART APEX CCD diffractometer	5087 independent reflections
Radiation source: sealed tube	4364 reflections with *I* > 2σ(*I*)
graphite	*R*_int_ = 0.039
φ and ω scans	θ_max_ = 25.8°, θ_min_ = 2.1°
Absorption correction: multi-scan (*SADABS*; Sheldrick, 1996)	*h* = −40→36
*T*_min_ = 0.305, *T*_max_ = 0.862	*k* = −12→12
14169 measured reflections	*l* = −11→19

### Refinement

**Table:**

Refinement on *F*^2^	Primary atom site location: structure-invariant direct methods
Least-squares matrix: full	Secondary atom site location: difference Fourier map
*R*[*F*^2^ > 2σ(*F*^2^)] = 0.031	Hydrogen site location: inferred from neighbouring sites
*wR*(*F*^2^) = 0.080	H-atom parameters constrained
*S* = 1.05	*w* = 1/[σ^2^(*F*_o_^2^) + (0.0425*P*)^2^] where *P* = (*F*_o_^2^ + 2*F*_c_^2^)/3
5087 reflections	(Δ/σ)_max_ = 0.002
379 parameters	Δρ_max_ = 1.72 e Å^−^^3^
0 restraints	Δρ_min_ = −0.59 e Å^−^^3^

### Fractional atomic coordinates and isotropic or equivalent isotropic displacement parameters (Å^2^)

**Table:**

	*x*	*y*	*z*	*U*_iso_*/*U*_eq_	
Ir1	0.365597 (4)	0.187358 (14)	0.693663 (9)	0.03540 (8)	
N1	0.32777 (11)	0.0250 (3)	0.6890 (2)	0.0416 (8)	
N2	0.39933 (11)	0.3598 (4)	0.7072 (2)	0.0426 (8)	
N3	0.38322 (11)	0.1393 (3)	0.5719 (2)	0.0401 (8)	
O1	0.41710 (9)	0.0620 (3)	0.71739 (17)	0.0441 (7)	
O2	0.45390 (11)	−0.0942 (4)	0.6590 (2)	0.0706 (10)	
O3	0.45584 (9)	−0.0840 (3)	0.4921 (2)	0.0564 (8)	
C1	0.33883 (15)	−0.1008 (4)	0.7107 (3)	0.0485 (11)	
H1	0.3663	−0.1186	0.7259	0.058*	
C2	0.31159 (19)	−0.2054 (5)	0.7119 (4)	0.0617 (14)	
H2	0.3203	−0.2935	0.7280	0.074*	
C3	0.2721 (2)	−0.1808 (5)	0.6897 (4)	0.0640 (16)	
H3	0.2531	−0.2520	0.6897	0.077*	
C4	0.25994 (15)	−0.0520 (5)	0.6672 (3)	0.0585 (12)	
H4	0.2324	−0.0342	0.6521	0.070*	
C5	0.28811 (13)	0.0521 (4)	0.6665 (3)	0.0441 (10)	
C6	0.28083 (15)	0.1936 (4)	0.6436 (3)	0.0453 (11)	
C7	0.31502 (13)	0.2809 (4)	0.6563 (3)	0.0409 (10)	
C8	0.30985 (14)	0.4155 (4)	0.6320 (3)	0.0503 (11)	
H8	0.3314	0.4776	0.6405	0.060*	
C9	0.27359 (16)	0.4575 (5)	0.5961 (3)	0.0582 (12)	
C10	0.24033 (16)	0.3774 (5)	0.5840 (3)	0.0613 (14)	
H10	0.2156	0.4105	0.5592	0.074*	
C11	0.24501 (13)	0.2484 (5)	0.6095 (3)	0.0536 (12)	
C12	0.41786 (14)	0.4204 (4)	0.6459 (3)	0.0521 (11)	
H12	0.4174	0.3770	0.5930	0.062*	
C13	0.43741 (16)	0.5417 (5)	0.6554 (4)	0.0663 (14)	
H13	0.4494	0.5824	0.6097	0.080*	
C14	0.43921 (18)	0.6028 (5)	0.7324 (4)	0.0722 (16)	
H14	0.4527	0.6865	0.7409	0.087*	
C15	0.42135 (16)	0.5422 (5)	0.7969 (3)	0.0639 (14)	
H15	0.4229	0.5834	0.8505	0.077*	
C16	0.40075 (13)	0.4197 (4)	0.7843 (3)	0.0458 (10)	
C17	0.37829 (14)	0.3462 (4)	0.8450 (3)	0.0446 (10)	
C18	0.35732 (12)	0.2308 (4)	0.8132 (3)	0.0378 (9)	
C19	0.33531 (14)	0.1565 (4)	0.8687 (3)	0.0434 (10)	
H19	0.3199	0.0808	0.8492	0.052*	
C20	0.33618 (17)	0.1936 (4)	0.9511 (3)	0.0525 (13)	
C21	0.35735 (19)	0.3014 (5)	0.9836 (4)	0.0683 (16)	
H21	0.3583	0.3225	1.0416	0.082*	
C22	0.37700 (17)	0.3773 (5)	0.9289 (3)	0.0649 (14)	
C23	0.43021 (13)	−0.0015 (4)	0.6533 (3)	0.0446 (10)	
C24	0.41319 (12)	0.0496 (4)	0.5690 (3)	0.0383 (9)	
C25	0.42684 (13)	0.0104 (4)	0.4908 (3)	0.0437 (10)	
C26	0.41040 (14)	0.0726 (5)	0.4191 (3)	0.0542 (12)	
H26	0.4201	0.0518	0.3663	0.065*	
C27	0.38003 (17)	0.1647 (5)	0.4247 (3)	0.0535 (13)	
H27	0.3682	0.2063	0.3756	0.064*	
C28	0.36687 (15)	0.1963 (4)	0.5008 (3)	0.0458 (11)	
H28	0.3457	0.2598	0.5040	0.055*	
C29	0.46854 (15)	−0.1295 (6)	0.4130 (3)	0.0619 (13)	
H29A	0.4832	−0.0568	0.3858	0.074*	
H29B	0.4450	−0.1555	0.3754	0.074*	
C30	0.49507 (17)	−0.2473 (7)	0.4297 (5)	0.0751 (17)	
H30	0.5047	−0.2911	0.3824	0.090*	
C31	0.5065 (2)	−0.2967 (5)	0.5026 (5)	0.080 (2)	
H31A	0.4976	−0.2565	0.5518	0.097*	
H31B	0.5237	−0.3731	0.5067	0.097*	
F1	0.27033 (10)	0.5876 (3)	0.5693 (2)	0.0866 (10)	
F2	0.21199 (9)	0.1647 (3)	0.5980 (2)	0.0759 (9)	
F3	0.31653 (10)	0.1165 (3)	1.00528 (17)	0.0748 (9)	
F4	0.39817 (14)	0.4857 (3)	0.9610 (2)	0.1073 (14)	

### Atomic displacement parameters (Å^2^)

**Table:**

	*U*^11^	*U*^22^	*U*^33^	*U*^12^	*U*^13^	*U*^23^
Ir1	0.03972 (12)	0.03992 (11)	0.02690 (11)	−0.00128 (6)	0.00483 (8)	−0.00145 (6)
N1	0.051 (2)	0.0460 (19)	0.0293 (19)	−0.0108 (16)	0.0137 (16)	−0.0091 (15)
N2	0.045 (2)	0.0455 (18)	0.038 (2)	−0.0053 (17)	0.0091 (17)	0.0043 (17)
N3	0.047 (2)	0.0424 (17)	0.0316 (19)	0.0009 (16)	0.0072 (16)	−0.0010 (16)
O1	0.0496 (17)	0.0548 (16)	0.0270 (15)	0.0049 (14)	−0.0033 (13)	0.0029 (14)
O2	0.085 (3)	0.074 (2)	0.053 (2)	0.038 (2)	0.0088 (19)	0.0119 (18)
O3	0.0536 (19)	0.0692 (19)	0.0477 (19)	0.0164 (16)	0.0118 (16)	−0.0069 (16)
C1	0.064 (3)	0.049 (2)	0.034 (2)	−0.008 (2)	0.009 (2)	−0.0010 (19)
C2	0.087 (4)	0.055 (3)	0.045 (3)	−0.016 (3)	0.013 (3)	−0.004 (2)
C3	0.078 (4)	0.065 (3)	0.051 (3)	−0.026 (3)	0.019 (3)	−0.009 (2)
C4	0.053 (3)	0.074 (3)	0.049 (3)	−0.019 (3)	0.009 (2)	−0.010 (3)
C5	0.046 (2)	0.058 (2)	0.030 (2)	−0.010 (2)	0.0149 (19)	−0.009 (2)
C6	0.042 (3)	0.061 (3)	0.034 (3)	0.002 (2)	0.008 (2)	−0.012 (2)
C7	0.045 (2)	0.051 (2)	0.028 (2)	0.0021 (19)	0.0061 (19)	−0.0085 (18)
C8	0.050 (3)	0.053 (2)	0.048 (3)	0.005 (2)	0.004 (2)	−0.009 (2)
C9	0.065 (3)	0.055 (3)	0.055 (3)	0.018 (2)	0.004 (3)	−0.011 (2)
C10	0.055 (3)	0.076 (3)	0.052 (3)	0.024 (3)	0.002 (2)	−0.016 (3)
C11	0.040 (3)	0.074 (3)	0.047 (3)	0.003 (2)	0.008 (2)	−0.015 (3)
C12	0.057 (3)	0.057 (3)	0.044 (3)	−0.010 (2)	0.015 (2)	0.008 (2)
C13	0.074 (4)	0.067 (3)	0.060 (3)	−0.021 (3)	0.016 (3)	0.013 (3)
C14	0.082 (4)	0.054 (3)	0.082 (4)	−0.025 (3)	0.011 (3)	0.003 (3)
C15	0.082 (4)	0.052 (3)	0.058 (3)	−0.022 (3)	0.010 (3)	−0.006 (2)
C16	0.055 (3)	0.042 (2)	0.040 (2)	−0.003 (2)	0.003 (2)	−0.0020 (19)
C17	0.056 (3)	0.045 (2)	0.034 (2)	−0.005 (2)	0.008 (2)	−0.0061 (19)
C18	0.042 (2)	0.0404 (19)	0.031 (2)	0.0027 (18)	0.0018 (18)	−0.0042 (18)
C19	0.050 (3)	0.049 (2)	0.032 (2)	−0.006 (2)	0.011 (2)	−0.0047 (19)
C20	0.067 (3)	0.055 (3)	0.038 (3)	−0.007 (2)	0.020 (3)	−0.003 (2)
C21	0.089 (4)	0.077 (4)	0.041 (3)	−0.016 (3)	0.019 (3)	−0.013 (3)
C22	0.097 (4)	0.055 (3)	0.044 (3)	−0.022 (3)	0.012 (3)	−0.020 (2)
C23	0.047 (3)	0.048 (2)	0.040 (3)	0.003 (2)	0.011 (2)	0.002 (2)
C24	0.042 (2)	0.0396 (19)	0.034 (2)	−0.0034 (18)	0.0066 (18)	−0.0004 (18)
C25	0.041 (2)	0.048 (2)	0.042 (3)	−0.0038 (19)	0.010 (2)	−0.005 (2)
C26	0.057 (3)	0.068 (3)	0.039 (3)	−0.006 (2)	0.013 (2)	−0.006 (2)
C27	0.062 (3)	0.071 (3)	0.027 (2)	0.007 (2)	0.000 (2)	0.003 (2)
C28	0.053 (3)	0.052 (2)	0.033 (2)	0.0068 (19)	0.004 (2)	−0.0005 (19)
C29	0.058 (3)	0.077 (3)	0.053 (3)	0.008 (3)	0.015 (3)	−0.021 (3)
C30	0.059 (3)	0.081 (4)	0.087 (5)	0.011 (3)	0.017 (3)	−0.022 (4)
C31	0.071 (4)	0.063 (3)	0.110 (6)	0.009 (3)	0.025 (4)	0.000 (3)
F1	0.090 (2)	0.0581 (16)	0.109 (3)	0.0262 (16)	−0.015 (2)	−0.0043 (17)
F2	0.0428 (16)	0.101 (2)	0.082 (3)	−0.0054 (15)	−0.0077 (16)	−0.0100 (18)
F3	0.100 (2)	0.087 (2)	0.0399 (16)	−0.0301 (18)	0.0257 (16)	−0.0026 (15)
F4	0.177 (4)	0.092 (2)	0.055 (2)	−0.071 (2)	0.024 (2)	−0.0337 (18)

### Geometric parameters (Å, °)

**Table:**

Ir1—C7	1.983 (4)	C12—C13	1.371 (6)
Ir1—C18	2.002 (4)	C12—H12	0.9500
Ir1—N1	2.045 (3)	C13—C14	1.373 (8)
Ir1—N2	2.051 (4)	C13—H13	0.9500
Ir1—N3	2.132 (3)	C14—C15	1.368 (7)
Ir1—O1	2.135 (3)	C14—H14	0.9500
N1—C1	1.339 (5)	C15—C16	1.404 (6)
N1—C5	1.375 (6)	C15—H15	0.9500
N2—C12	1.341 (5)	C16—C17	1.465 (6)
N2—C16	1.367 (6)	C17—C22	1.384 (6)
N3—C24	1.343 (5)	C17—C18	1.417 (6)
N3—C28	1.352 (6)	C18—C19	1.404 (6)
O1—C23	1.307 (5)	C19—C20	1.369 (7)
O2—C23	1.212 (5)	C19—H19	0.9500
O3—C25	1.346 (5)	C20—F3	1.361 (5)
O3—C29	1.438 (6)	C20—C21	1.365 (7)
C1—C2	1.381 (7)	C21—C22	1.359 (7)
C1—H1	0.9500	C21—H21	0.9500
C2—C3	1.364 (9)	C22—F4	1.367 (5)
C2—H2	0.9500	C23—C24	1.514 (6)
C3—C4	1.380 (7)	C24—C25	1.419 (6)
C3—H3	0.9500	C25—C26	1.383 (6)
C4—C5	1.398 (6)	C26—C27	1.373 (7)
C4—H4	0.9500	C26—H26	0.9500
C5—C6	1.465 (6)	C27—C28	1.362 (7)
C6—C11	1.391 (7)	C27—H27	0.9500
C6—C7	1.436 (6)	C28—H28	0.9500
C7—C8	1.397 (6)	C29—C30	1.479 (8)
C8—C9	1.369 (7)	C29—H29A	0.9900
C8—H8	0.9500	C29—H29B	0.9900
C9—F1	1.360 (5)	C30—C31	1.299 (10)
C9—C10	1.369 (7)	C30—H30	0.9500
C10—C11	1.349 (8)	C31—H31A	0.9500
C10—H10	0.9500	C31—H31B	0.9500
C11—F2	1.382 (5)		
			
C7—Ir1—C18	90.86 (17)	C13—C12—H12	118.2
C7—Ir1—N1	81.10 (16)	C12—C13—C14	118.5 (5)
C18—Ir1—N1	94.43 (15)	C12—C13—H13	120.7
C7—Ir1—N2	95.32 (15)	C14—C13—H13	120.7
C18—Ir1—N2	80.32 (16)	C15—C14—C13	119.4 (5)
N1—Ir1—N2	173.64 (13)	C15—C14—H14	120.3
C7—Ir1—N3	96.53 (15)	C13—C14—H14	120.3
C18—Ir1—N3	171.88 (15)	C14—C15—C16	120.5 (5)
N1—Ir1—N3	90.06 (13)	C14—C15—H15	119.7
N2—Ir1—N3	95.59 (14)	C16—C15—H15	119.7
C7—Ir1—O1	170.11 (14)	N2—C16—C15	119.3 (4)
C18—Ir1—O1	96.88 (14)	N2—C16—C17	113.4 (3)
N1—Ir1—O1	92.13 (13)	C15—C16—C17	127.2 (4)
N2—Ir1—O1	92.06 (13)	C22—C17—C18	118.7 (4)
N3—Ir1—O1	76.16 (12)	C22—C17—C16	126.0 (4)
C1—N1—C5	119.6 (4)	C18—C17—C16	115.2 (4)
C1—N1—Ir1	124.4 (3)	C19—C18—C17	117.6 (4)
C5—N1—Ir1	116.0 (3)	C19—C18—Ir1	127.5 (3)
C12—N2—C16	118.7 (4)	C17—C18—Ir1	114.8 (3)
C12—N2—Ir1	125.1 (3)	C20—C19—C18	119.7 (4)
C16—N2—Ir1	116.1 (3)	C20—C19—H19	120.2
C24—N3—C28	120.4 (4)	C18—C19—H19	120.2
C24—N3—Ir1	115.8 (3)	F3—C20—C21	117.2 (5)
C28—N3—Ir1	123.8 (3)	F3—C20—C19	119.1 (4)
C23—O1—Ir1	116.9 (3)	C21—C20—C19	123.6 (5)
C25—O3—C29	117.5 (4)	C22—C21—C20	116.6 (5)
N1—C1—C2	122.2 (5)	C22—C21—H21	121.7
N1—C1—H1	118.9	C20—C21—H21	121.7
C2—C1—H1	118.9	C21—C22—F4	117.0 (5)
C3—C2—C1	119.3 (5)	C21—C22—C17	123.7 (4)
C3—C2—H2	120.4	F4—C22—C17	119.3 (5)
C1—C2—H2	120.4	O2—C23—O1	124.1 (4)
C2—C3—C4	119.7 (5)	O2—C23—C24	121.4 (4)
C2—C3—H3	120.2	O1—C23—C24	114.5 (4)
C4—C3—H3	120.2	N3—C24—C25	120.0 (4)
C3—C4—C5	119.9 (5)	N3—C24—C23	115.1 (4)
C3—C4—H4	120.0	C25—C24—C23	124.9 (4)
C5—C4—H4	120.0	O3—C25—C26	124.4 (4)
N1—C5—C4	119.4 (4)	O3—C25—C24	117.0 (4)
N1—C5—C6	113.1 (4)	C26—C25—C24	118.6 (4)
C4—C5—C6	127.5 (4)	C27—C26—C25	119.6 (4)
C11—C6—C7	118.3 (4)	C27—C26—H26	120.2
C11—C6—C5	126.5 (4)	C25—C26—H26	120.2
C7—C6—C5	115.2 (4)	C28—C27—C26	120.0 (5)
C8—C7—C6	117.0 (4)	C28—C27—H27	120.0
C8—C7—Ir1	128.1 (3)	C26—C27—H27	120.0
C6—C7—Ir1	114.4 (3)	N3—C28—C27	121.4 (4)
C9—C8—C7	119.8 (4)	N3—C28—H28	119.3
C9—C8—H8	120.1	C27—C28—H28	119.3
C7—C8—H8	120.1	O3—C29—C30	107.4 (5)
F1—C9—C8	118.0 (5)	O3—C29—H29A	110.2
F1—C9—C10	117.3 (4)	C30—C29—H29A	110.2
C8—C9—C10	124.6 (5)	O3—C29—H29B	110.2
C11—C10—C9	115.6 (4)	C30—C29—H29B	110.2
C11—C10—H10	122.2	H29A—C29—H29B	108.5
C9—C10—H10	122.2	C31—C30—C29	126.5 (6)
C10—C11—F2	117.0 (4)	C31—C30—H30	116.7
C10—C11—C6	124.6 (5)	C29—C30—H30	116.7
F2—C11—C6	118.4 (5)	C30—C31—H31A	120.0
N2—C12—C13	123.5 (5)	C30—C31—H31B	120.0
N2—C12—H12	118.2	H31A—C31—H31B	120.0

### Hydrogen-bond geometry (Å, °)

**Table:**

*D*—H···*A*	*D*—H	H···*A*	*D*···*A*	*D*—H···*A*
C3—H3···F1^i^	0.95	2.60	2.997 (6)	106
C28—H28···F2^ii^	0.95	2.55	3.280 (6)	134
C10—H10···F3^iii^	0.95	2.50	3.299 (6)	142
C12—H12···F4^iv^	0.95	2.56	3.130 (6)	119
C26—H26···O1^v^	0.95	2.63	3.519 (5)	155
C14—H14···O2^vi^	0.95	2.54	3.275 (6)	134

Symmetry codes: (i) *x*, *y*−1, *z*; (ii) −*x*+1/2, −*y*+1/2, −*z*+1; (iii) −*x*+1/2, *y*+1/2, −*z*+3/2; (iv) *x*, −*y*+1, *z*−1/2; (v) *x*, −*y*, *z*−1/2; (vi) *x*, *y*+1, *z*.
